# Ecological urbanism as a condition underpinning urban life for internally displaced peoples in Colombia

**DOI:** 10.1371/journal.pone.0291794

**Published:** 2023-09-19

**Authors:** Paula Andrea Valencia Londoño, Diana Valencia Londoño, Phoenix Storm Paz

**Affiliations:** 1 Faculty of Social and Human Sciences, University of Medellín, Medellín, Antioquia, Colombia; 2 Faculty of Integrated Arts, University of San Buenaventura, Medellín, Antioquia, Colombia; Gebze Teknik Universitesi, TURKEY

## Abstract

This paper employs the concepts of sustainability and habitability to define and measure ecological urbanism in informal settlements founded by people who have been forcibly displaced by violence. The objective is to identify the obstacles in meeting the tenets of ecological urbanism in informal settlements. We offer primary research from La Primavera, an informal settlement founded by internally displaced people in the late 1970s, located in the *corregimiento* El Hatillo of Barbosa, Antioquia in northwestern Colombia. Primary research includes qualitative and quantitative data gathered through 72 surveys and 45 technical assessments of properties and houses in the settlement. Situated between the Aburrá River and the northbound highway, above a gas pipeline and under an electricity pylon, La Primavera exists in a state of extreme risk, exacerbated by increasing migration which threatens to exceed the carrying capacity of the territory. Moreover, in Article 35 of Law 388 of 1997, the territory was declared a protected or restricted area and earmarked for the development of the regional commuter train, called the *Tren de Cercanías*, in 2017. The case study highlights the contradictions in the development agenda in Colombia by showing how development projects designed for the economic betterment and environmental conservation of the region negatively impact the quality of life for the most vulnerable inhabitants and expose them to greater environmental, economic, and social risk.

## 1. Introduction

UN-Habitat [1] states that “informal settlements, marginal neighborhoods, and other poor residential neighborhoods are an urban phenomenon worldwide,” and Latin America is the epicenter of this problem. According to estimates by the Economic Commission for Latin America and the Caribbean (ECLAC), one in every five people in the region live in informal settlements in conditions of poverty, and these informal settlements embody what ECLAC [2] calls the three contradictions of Latin America: economic growth marked by the persistence of inequality and poverty; reduction in housing deficits but with spatial, social, and economic segregation; and democratization with high indices of violence. According to the World Bank, seven of the 15 countries exposed to three or more natural threats are located in Latin America and the Caribbean [3]. Predictions for the future in Latin America are not encouraging. In the short term, a qualitative deficit in housing is expected, and, in the long-term, an increase in informal settlements that are the products of the reduction of house size, separation of families, and the increase in life expectancy [4–7].

In the case of Colombia, the persistence of phenomena like forced internal displacement and the reception of international migration exacerbate the problems of informal settlement and precarity along the peripheries of urban areas. The city of Medellín and its surrounding metropolitan area are no exception. Growth and urban consolidation in Medellin since the end of the nineteenth century through the beginning of the twenty-first century have been characterized by densification along the periphery, the result of the arrival of economic migrants motivated by opportunities offered in the city as well as populations displaced by violence, exiled from their home territories in the constant episodes of rural violence that the country has experienced.

The institutional and private response to migration to and within the city has been insufficient, resulting in the consolidation of an important number of precarious settlements on the periphery of the city with origins that go as far back as the 1950s. In the decade between 1950 and 1960, Medellin doubled its population, and new inhabitants founded settlements in the most inaccessible areas surrounding Medellin, places that are both environmentally complex and fragile. These informal settlements are pejoratively called pirate urbanizations, or invasions, and are characterized by self-construction, the inexistence of public service infrastructures, and illegality [8,9].

Law 388 of 1997 [10] regulates territorial development and management plans at the municipal level. Articles 11 and 12 of the law determine which areas are critical for the prevention of natural disasters, the realization of conservation goals, and landscape recuperation. Moreover, articles 11 and 12 identify environmentally important ecosystems and establish the norms for their protection and management, which permits the incorporation of these ecosystems into the municipal urban ecological structure as “protected lands,” at least in the spirit of the law. Nonetheless, in Medellin, past and present urban development projects have been carried out on these declared protected lands despite the law. This article highlights how the process of accelerated urbanization has increased the presence of high-risk zones. Managing the environmental risk generated by the fragility of the occupied territories has become a goal for urban planners who choose a paradigm of ecological urbanism because the risks disproportionately affect the most impoverished populations of the city–populations that continuously struggle against precarious conditions of habitability. Therefore, the objective of this paper is to identify the obstacles to ecological urbanism, read from the perspective of habitability, in informal settlements founded by people forcibly displaced by violence and conflict. We ground the discussion in the case study of La Primavera in Barbosa, Colombia. We begin by explaining our methodology and materials. Then, we describe the site of the case study before moving onto the theoretical background of the study, in which we define habitability and sustainability as two necessary elements of ecological urbanism. We suggest that sustainable construction is the way in which these concepts are physicalized in architecture and urban design. Using the criteria set in the discussion of sustainable construction, we evaluate the results from the case study of La Primavera. We close with a holistic analysis of the development agenda in Colombia and ask where informal settlements like La Primavera fall within this agenda.

## 2. Materials and methods

In this article, we seek to identify the components that satisfy the criteria of sustainable construction, both theoretically as a part of the means of evaluating ecological urbanism, and practically, as a means of understanding living conditions in the site our case study: La Primavera. We thus employ an empirical-analytical research paradigm, as defined by Vasco [11], who argues that the purpose of academic research is social transformation. Analytical-empirical analysis assesses the empirical, observable world to identify and deconstruct problems in order to propose solutions or alternatives [11]. In the research presented below, we evaluate the conditions of habitability in the informal settlement of La Primavera to identify problems and uncover the underlying systems that permit such conditions to continue to exist. Thus, our research is empirical in the approach used to evaluate the physical conditions of habitability and sustainability in La Primavera but analytical in aim: to understand how the tenets that underlay the concept of sustainable construction are met, or not, in contexts of informal settlements made by the forcibly displaced migrant population.

In terms of approach, Bryman [12] argued that researchers should not adhere to one method in particular but rather choose the method most appropriate to the questions being asked and the techniques pertinent to the objectives of the research, his famous “technical argument.” Further, Marradi, Archenti, and Piovani that “all acts of empirical investigation involve a combination of quality and quantity” [13]. We thus consider qualitative and quantitative methods to be both compatible and equally valid. Based on this reasoning, we have chosen to employ a triangulation methodology in which two or more methods are used in the research process “to approach the same problem, or different but closely related problems,” also known as multimethod research.

Our objective in this study is to uncover the relationship between micro and macro processes. One the one hand, we wish to evaluate the extent to which the criterion of sustainable construction are met, or not, within La Primavera, the chosen case study site. On the other hand, we seek to understand the determinants of sustainable construction from its macro structural level derived from the ideological commitments of ecological urbanism. Due to our two-part objective, we employed a triangulation methodology in the tradition of Bryman [11] and Marradi, Archenti, and Piovani [12], which mobilizes qualitative and quantitative techniques. The chosen research method follows a deductive-inductive-deductive model, in which we first take a theoretical approach to the criteria of measuring sustainable construction in order to later present a descriptive analysis of the case. This description combines qualitative and quantitative analysis to measure the extent to which the criteria of sustainable construction are met in the informal settlement of La Primavera, as a means of measuring ecological urbanism, understood as the conjunction of internal and external habitability and sustainability. It is the product of the research program “Vulnerability, resilience and risk of communities and supplying basins affected by landslides and avalanches” code 1118-852-71251, contract 80740–492–2020 between Fiduprevisora and the University of Medellin with resources from Minciencias: The National Financing Fund for Science, Technology and Innovation—Francisco José de Caldas Grant. This research program and the instruments included within were evaluated and approved by the Committee of Ethics of Research at the University of Medellin though Act No. 294 of August 20, 2019, prior to applying for financial support. Both the qualitative and quantitative analyses focused on occupant comfort, access to facilities, participation and control, education, health and safety, as well as adaptability and flexibility of the sites and the management of water and waste as the primary indicators in internal and external habitability and environmental sustainability.

Gouin et al. [14] stipulated that the process of environmental risk analysis should include the following components: the quantitative assessments of health or ecosystem risk [15] and the communication and management of that risk [16]. In the risk assessment stage, different instruments have been incorporated, such as internal and external habitability sheets that allow researchers to determine the environmental risks to which communities are exposed as well as the risks that derive from the condition of their environment and families due to the conditions present inside the house.

Prior to the application of the instruments, participants were clearly and truthfully informed about the research project, its objectives, procedures, and risks, and gave their consent in writing. Moreover, they acted in a voluntary and conscious manner during the collection of information, which included the realization of surveys, the application of psychometric tests, interviews, and workshops/focus groups, all of which recorded. All participants were informed at every step of the research that they could abstain from responding to any or all the questions, partially answer any question, or retire from the process at any time without need for justification. Participation was voluntary, unpaid, and limited to individuals above 18-years-old, the age of majority. (To see survey results, please consult S2 File Dataset 1. Survey Responses.)

Of the three criteria of social, economic, and environmental sustainability planted by Reffat [17] regarding sustainable construction, we evaluated two in the following study of La Primavera: social and environmental sustainability. We have chosen not to address the economic elements here because they don’t play into the theoretical definition of internal and external habitability, the means of measurement that we have chosen to evaluate ecological urbanism. To measure internal and external habitability, we used a mixed methodology that implicated a combination of qualitative and quantitative methodologies.

The qualitative analysis entailed the application of a large-scale household survey consisting of 58 questions structured along the following axes of analysis: time lived in the territory, permanence of the structure, location of the structure, conditions of tenancy of the household, threat and risk, overcrowding, use, interior comfort, access to public services and amenities in the house. Questions were either multiple-choice in which respondents could select one or more answers, or open-ended questions in which respondents could reply in as much detail as desired. (See the S1 File La Primavera Habitability Interview Questionnaire at the end of the document for a copy of the interview questions.) During analysis, questions were grouped in three categories (internal habitability, external habitability, and environmental risk) with various attributes describing each category. Each attribute could receive a grade of high, medium, or low quality. (See Table 1 for further detail.)

**Table 1:** Categories, variables, and indicators of measurement.

Category	Description of Variable or attribute	Indicator and Quality Level	Measurement
Internal Habitability	Construction Materials: Premise—the materials used should be safe, responsive to environmental conditions, and economically accessible for residents.	Structural System: Porticoes: High Masonry: Medium Stilt house: Low Walling Material: Brick or cement block: High Tapia: Medium Wood: Low Roofing Material: Losa: Medium Clay roof tiles: High Asbestos Cement / Zinc: Low Flooring Material: Tile: High Cement mortar: Medium Dirt: Low	In relation to construction materials, greater weight was assigned to the materials associated with the structural systems. The weight assigned to the construction materials was: 40% structure, 20% walls, 20% roofing, 20% flooring. High: 5 points Medium: 3 points Low: 2 points Internal Habitability Construction Materials = (structure * .4) + (walling * 0.2) + (roofing * 0.2) + flooring * 0.2) Note: the weighting system gives a greater value to the structural system because house structure protects the population from physical vulnerability, fulfilling the established requirements established by the Colombian code for seismic resistant construction NSR10. The other materials support internal habitability because they grant greater comfort.
	House size: The adequacy of house size was determined according to the minimum size established by the legal norm for social living in Colombia. Houses do not necessarily have to be large, but they should have enough room that all residents can carry out their daily tasks without feeling confined. Moreover, if the building is a multi-use structure, the ideal condition is that there is enough space for the different uses to remain separate.	Minimum legally permitted house size: 42 m^2^ Minimum legally permitted lot size: 30 m^2^ Area of House > 70m^2^: High 42m^2^ < Area of House < 70m^2^: Medium Area of House < 42m^2^: Low	House Size (HS): 40% Overcrowding (OC): 30% Population Density (PD): 30% Internal Habitability = (HS * 0.4) + (OC * 0.3) + (PD * 0.3)
	Population Density seeks to identify conditions of overcrowding in the house. The Colombian legal norm for social living establishes that a house with the minimum area required by law (42m^2^) should be home to a single family unit. To have more than one family living in the minimum legally sized home is considered overcrowding.	Overcrowding: # family/house >1.0: low No overcrowding: # families = # houses	
	Number of people that inhabit the house: divide the minimum area of the house by the average number of people that make up the family unit.	# m^2^ per person / house > 10 m^2^/person: High 10 m^2^/person: Medium < 10 m^2^/person: Low	
	Access to public services.	Energy Water Waste collection Sewage	High: Respondents have access to 3 or 4 public services Medium: Respondents have access to 2 public services Low: Respondents have access to 0 services or 1 public service
	Perception of the suitability of the residence for one’s everyday needs.	The spaces in the house are enough to fulfil the needs of the family unit.	High: Yes Low: No
External Habitability We have argued that habitability goes beyond seeing the building as an individual unit, and that to truly analyze if a home is habitable, one must understand that a house is situated in a city network. The network must be navigable, and public services like education and healthcare must be available, as well as access to places to buy food and other basic necessities and to social facilities.	Provision of public facilities	Number of facilities located close to the settlement.	High: Respondents that can identify 4 or 5 public facilities close to their house Medium: Respondents that can identify 2 or 3 public facilities close to their house Low: Respondents that identify 0 or 1 facility close to their house
Environmental RiskEnvironmental risk is defined as the possibility that an event with grave environmental consequences occurs. Environmental risk, therefore, refers to the final impact and not to the event that caused it. The GTC 104 in 2009 –Environmental Risk Management and the European model UNE 15008 (2008)–defines environmental risk as resulting from a function that relates the probability of occurrence of a specific type of accident and the negative consequences of the same on the natural, human, and socioeconomic environment. The analysis of risk is comprised of two parts: • Definition of the types of causal accidents • Definitions of the types of consequences The connection between the two are the event indicators, which are the physical facts generated by the causal event that generates the consequences[40].	Risk: The right to a dignified residence guaranteed in Article 51 of the 1991 Political Constitution of Colombia requires that the places where people live be safe for habitation and environmentally sustainable. Therefore, Law 338 of 1997 was allowed to declare certain lands restricted because the level of non-mitigable environmental risk was too high for safe settlement or because human occupation exceeds the carrying capacity of the territory.	Risk of Landslide	High: (% perception of risk of landslide + % risk of the location of landslide) /2 Medium: (%perception of risk + % risk of the location) /2 Low: (%perception of risk + % risk of the location) /2
		Risk of Flooding	High: (%perception of risk of flooding + % risk of flooding at the location) /2 Medium: (%perception of risk of flooding + % risk of flooding at the location) /2 Low: (%perception of risk of flooding + % risk of flooding at the location) /2
		Risk of Sinking	High: (%perception of risk sinking + % risk of sinking at the location) /2 Medium: (%perception of risk sinking + % risk of sinking at the location) /2 Low: (%perception of risk sinking + % risk of sinking at the location) /2

Surveys were applied to an initial base of 72 heads of household, chosen from a total of 183 households identified in the September 2022 census. However, not every question on the survey was answered by all 72 respondents. For the statistical analysis, the “no-response” or “decline to respond” answers were discarded. As a result, not every question has the same total number of responses, and the total used for the statistical analysis is given for each question. The sample is described in Table 2.

**Table 2:** Description of the sample.

Individuals Interviewed (Total of 72 responses used for calculation)	Number of respondents		Percentage
	Women	46	63.9%
	Men	25	34.7%
	Declined to respond	1	1.4%
Age of the individual interviewed (Years)	Average (Mean)	48	--
	Minimum	18	--
	Maximum	81	--
Length of time the individual has lived in the neighborhood (Years)	Average (Mean)	14	--
	Minimum Time	2 months	--
	Maximum Time	40 years	--
Sector (Total of 72 responses used for calculation)	Alto	28	38.9%
	Medio	27	37.5%
	Bajo	17	23.6%
Legal Status of Residency (Total of 72 responses used for calculation)	Proprietor	44	61.1%
	Renter	13	18.1%
	Possessor	12	16.7%
	Family inheritor	1	1.4%
	Squatter	1	1.4%
	House Sitter	1	1.4%

For the quantitative analysis, the results of the sample were segmented according to variables, and the measurement described above was carried out on each of the indicators that composed the analysis. We also conducted a schematic survey of the interior distribution of the house accompanied by a photographic record of 45 homes in La Primavera. Furthermore, the quantitative data was complemented by a qualitative model for evaluating the habitability of each residence: technically trained personnel, including architects and engineers, evaluated the infrastructure conditions and environmental threat and risk for each of the selected residences in the settlement.

For the qualitative component, observation was conducted in each of the public spaces and facilities, in which the state, the traffic, and the use of each space was evaluated. These observations were conducted by different researchers on the team during various field research trips. The results are a synthesis of the external habitability conditions (including education and healthcare, community spaces, and public spaces, among others) (See S3 File. Dataset 2. Example Public Space or Facility Observation Card). Finally, a social mapping workshop was carried out with the inhabitants of the settlement in which they identified the factors of natural and anthropic risk or threat and the origin or sources of environmental contamination. Said attributes were located on the map, and their causes were synthesized in summaries provided in the discussion carried out for each table.

## 3. The settlement of La Primavera

**Fig 1:**
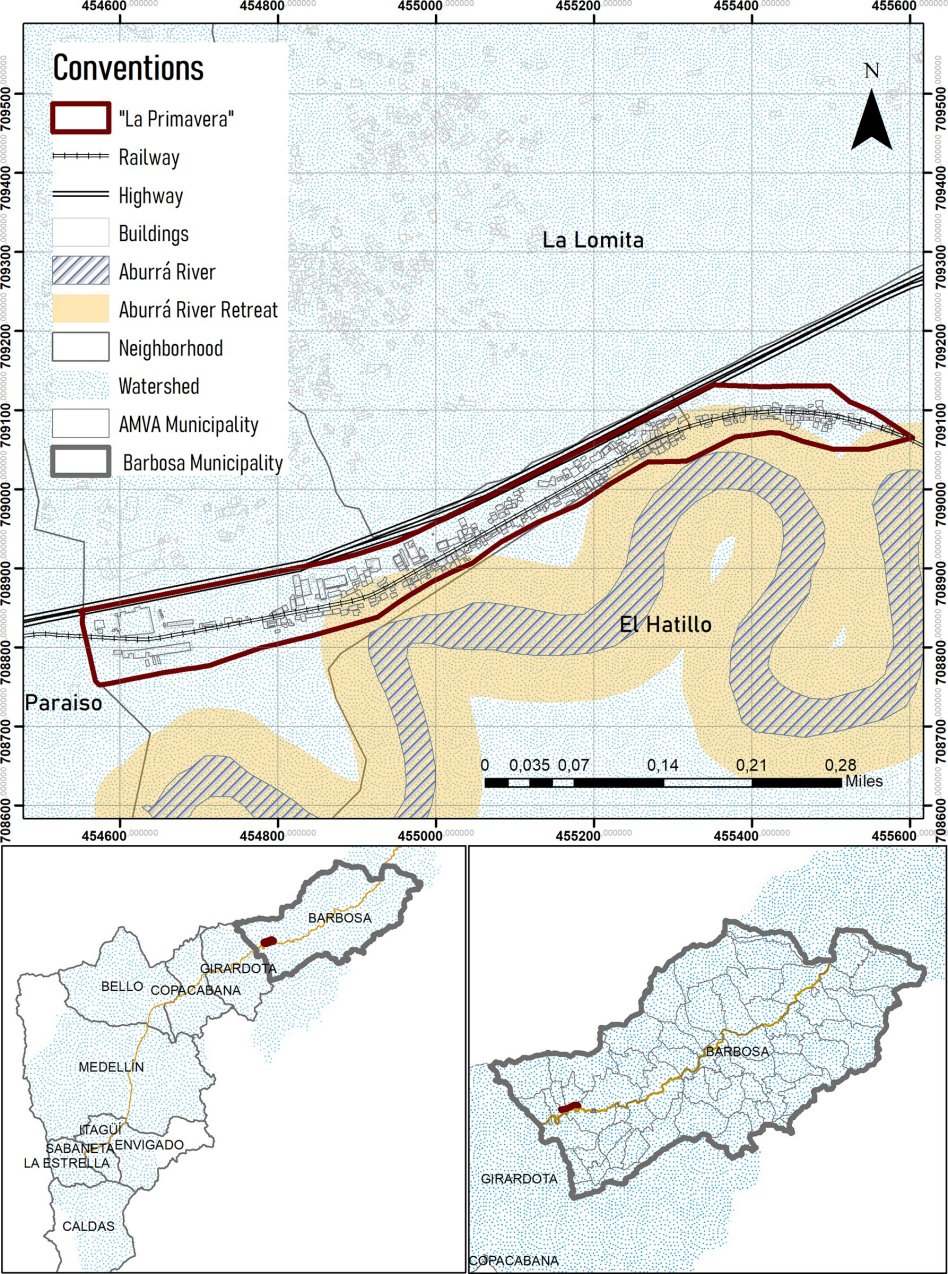
Map of the La Primavera settlement in the municipality of Barbosa. Source: Own preparation.

La Primavera, the focus of this case study, is an informal settlement in the Valley of Aburrá. Located approximately 42km north of Medellin, in the municipality of Barbosa, La Primavera is a peri-urban settlement situated in the *corregimiento* of El Hatillo along the northern bank of the Aburrá River. Fig 1 gives a map of La Primavera. The bottom left of the map delineates the boundaries of the municipality of Barbosa, and the bottom right highlights the location of the settlement within the municipality. The top image of the map shows the distribution of the settlement along the riverbank. The different sectors of the settlement, called Alto, Medio, and Bajo by residents, are marked in red; Alto is the oldest sector, while Bajo is the newest. As can be seen in the map, Bajo in the east of the settlement is located closest to the Aburrá River, where most of the houses in the sector are situated within the flood zone of the riverbank.

The lands on which La Primavera is settled have been declared a protected or restricted area under Article 35 of Law 388 of 1997. Under said law, lands can be declared protected areas or non-intervention areas due to their geographic, landscape, or environmental characteristics, earmarked as zones for infrastructure or public utility development, or declared restricted due to high, non-mitigable risk to human settlement [10]. No matter the specific classification of the area, housing development and urbanization is severely limited or outright prohibited. In La Primavera, the territory is declared protected because it meets all three conditions of the law. The settlement extends all the way down to the riverbank, which is considered an at-risk natural habitat, and the protection of the waters of the Aburrá River is of grave concern to local and national conservation efforts alike. Furthermore, there has been substantial development and intervention in the landscape for the provision of public utilities, and La Primavera is situated above a gas pipeline, under an electricity pylon, alongside the northbound highway over the historic train tracks. Finally, the combination of natural landscape features and public infrastructure interventions means the area is substantially affected by flooding and landslides, both of which are non-mitigable risks that put human settlers in danger.

**Fig 2:**
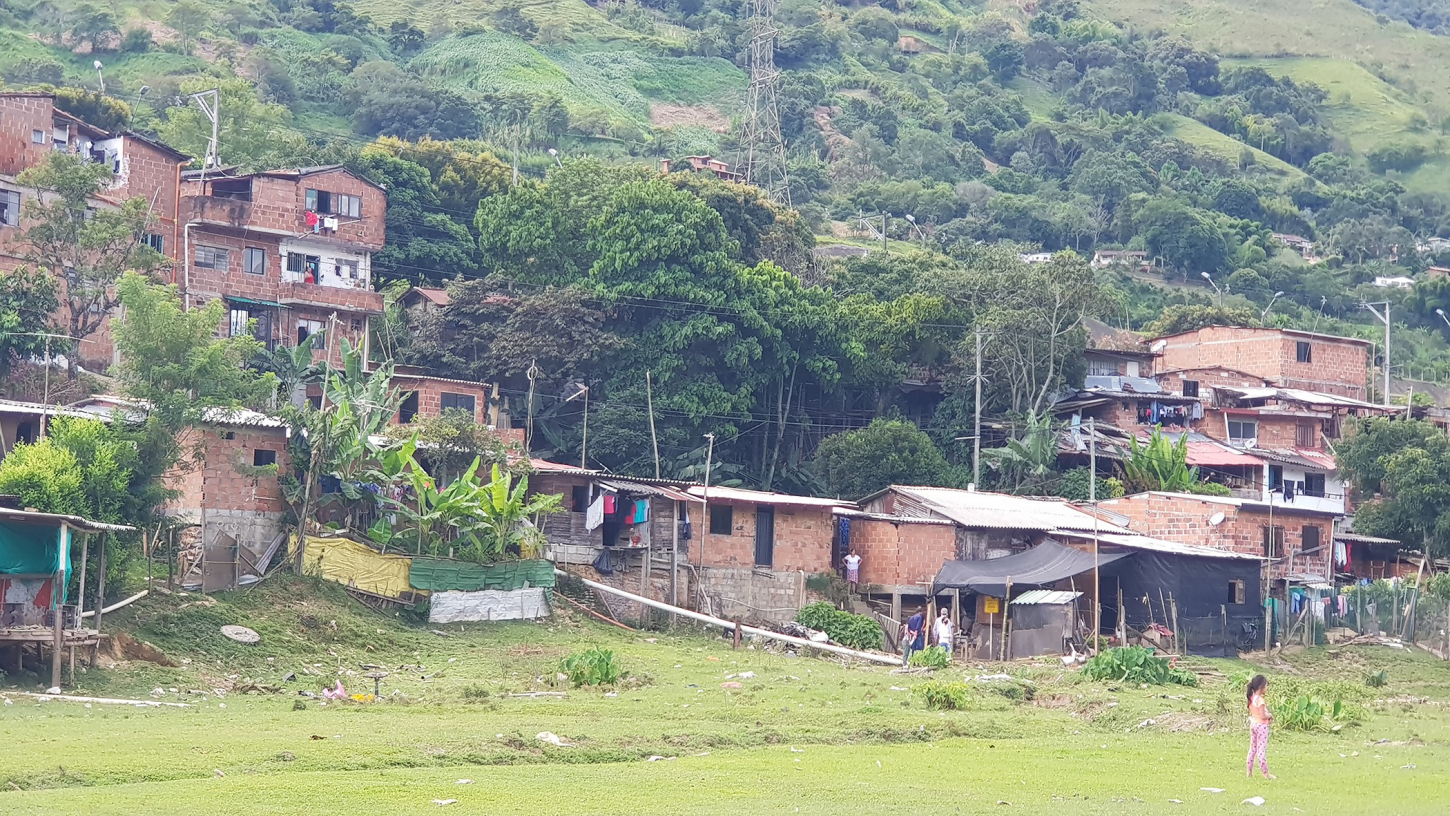
Photograph of La Primavera settlement. Source: Photograph taken by the authors.

Thus, the settlement is in a state of existential crisis: the social situation caused first by the Armed Conflict, then by the drug trade, and most recently by internal urban displacement has pushed people to the area for decades, while the built and natural environmental conditions make it an untenable place to live. However, to understand why La Primavera was situated in its current location despite the protected or restricted legal status of the land, it must be understood that the settlement was founded in 1978, long before Law 388 was passed, by people forcibly displaced from the Chocó and Urabá regions of Antioquia by the Armed Conflict [18]. Fig 2 shows a picture of the settlement taken by the authors during their field research in 2021.

The settlement’s history can be divided into four periods. The first period spans from the settlement’s founding in the late 1960s and 1970s until 2001. During this time, the territory was populated with a growing number of refugees, and the settlement expanded downwards towards the river. In 2001, the settlement was massacred [18]. The second period of La Primavera’s history, which lasted from 2002 to 2013, can be considered the period of consolidation. In 2013, residents created the Association of Victims and Displaced Persons *Los Meadros Nuevo Amanecer* [18].

Growth is the defining characteristic of La Primavera’s third historical period which stretched from 2013 to 2018 [18]. In 2016, the Department of Antioquia in conjunction with the Municipal Government of Medellin and the Metropolitan Area of the Valley of Aburrá announced that construction of the local commuter train to connect all 11 municipalities in the 83km-long valley would begin upon the completion of the feasibility assessments scheduled for 2017 [19]. The promise of stable employment and economic growth brought new waves of migrants from rural regions and the peripheries of larger cities in the Metropolitan Area [18] where insecurity and impoverishment are high, and residents fear urban organized crime and other illegal armed actors.

The last study of the settlement was conducted in 2015 by Corporación Región in partnership with the University of Antioquia, financed by the Iglesia Católica Alemana Misereor, a local NGO, when the number of inhabitants in the settlement totaled 570 people [18]. Of these, 392 were individuals forcibly displaced and legally registered as Victims of the Armed Conflict in the *Registro Unico de Victimas* (The National Registry of Victims). Almost half, 49.6%, of the families resettled in La Privavera were displaced from other municipalities in the Department of Antioquia, including Anorí, El Bagre, La Pintada, Ituango, San Jerónimo, Itango-Vereda El Aro, Segovia, Taraza, Cereté. The rest of the population was comprised of second and third generation of inhabitants, children or grandchildren of the displaced population, as well as newly arrived migrants who are not legally classified as Victims. The second largest percentage of the population, for instance, is comprised of people forcibly displaced from the cities in the Metropolitan Area of the Valley of Aburrá (Bello, Barbosa, Itagüí, and neighborhoods in Medellin like Zamora, Santo Domingo, and La Comuna 13), but who do not fall into the legal category of Victims of the Armed Conflict. In many cases, many of these people came to live in La Primavera after other processes of displacement. Only 4.3% of the population comes from other departments in the country [19]. Data regarding length of residency confirms the settlement pattern. Individuals recognized as Victims in the 2015 census had an average of 18.3 years of residency in La Primavera, in contrast with those classified as economic migrants or urban refugees who had, on average, less than 8 years of residency in the settlement [18].

The last period in La Primavera’s history spans from 2018 until the present day. From the 83 residences recorded by Corporación Región in 2015, the settlement grew to 168 residences by 2018, and continues to grow at unprecedented rates [18]. Recently, internal urban displacement has become an important dynamic in the municipality of Barbosa. Moreover, the city has received ever growing numbers of international refugees fleeing the economic collapse in Venezuela. The promise of affordable housing has drawn many of these newly arrived migrants to La Primavera and other similar settlements [18]. Tensions between the different population groups have increased due to competition for resources and the absence of the state in the territory, and as different bands of armed actors have stepped in to take the role of absent state [18]. Furthermore, the rapid increase in settlement along the riverbank and the exploitation of the river’s resources have undermined the stability of the land along the river’s edge, causing erosion that eats away at the foundations of many of the settlement’s residences [18]. In short, the unprecedented population growth has increased both the social and the environmental risk experienced by residents, leading to new concerns regarding sustainability and the long-term viability of the settlement, and raised the possibility of relocation as a matter of necessity.

The questions of social and ecological instability are not the only causes for concern. Recent plans for the local commuter train, a project that promised to bring economic prosperity to the region and drew people to the settlement, have been published, and planners have announced their intention to reuse the pre-existing train tracks as the basis for the new transport infrastructure [20]. For residents of La Primavera, that means the new train will go right through the middle of their homes, requiring that they move before construction begins. At the moment, it is unclear if the state will offer assistance in relocating residents, or buy their houses at market value. Furthermore, as the settlement is still informal, the market value of the homes that will be destroyed by the project is undetermined.

La Primavera is not the only informal settlement facing an uncertain future. The sad fact is that there is no formal process to regulate the consolidation and incorporation of informal settlements, nor the treatment of the people who live in such places. For decades, urban and regional planning in Colombia has been characterized by the absence of directives to guide territorial settlement and establish requirements for settlements. Only in 2015 did the Colombian national government adopt Objective 11 of United Nation’s 2030 Agenda for Sustainable Development “Sustainable Cities and Communities” into regional planning, requiring that planners and developers include environmental determinants as a primary criterion for the development of a territory for human settlement. The adoption of Objective 11 means that urban and regional planners must address the environmental problems that result from informal settlements as well as establishing a basic quality of life for rural-urban migrants in these settlements. Nonetheless, planners lack the theoretical and practical instruments to evaluate quality of life in and the sustainability of new settlements.

## 4. Ecological urbanism: The conjunction between habitability and sustainability

### 4.1 Ecological urbanism: Reconceiving the city as an ecosystem

“[Cities] consume energy in their operation and in the extraction and preparation of materials, and they generate waste and pollution,” argued Egyptian architect Rabee M. Reffat [17] in the *First International Architecture Conference* of Cairo University in 2004. In short, cities are like living organisms; they have a “metabolism” [21] in that they consume resources and produce waste. Thus, like all other organisms, cities are part of a larger system, an ecosystem, and can only survive if their surrounding ecosystems do as well.

The concept of understanding cities as organisms and therefore designing them in balance with the natural environment was first proposed by architect and planner Miguel Ruano who introduced the concept of “EcoUrbanism,” or “the development of multi-dimensional sustainable human communities within harmonious and balanced built environments” to scholars in 1998 [22]. In the early twenty-first century, the concept underwent a period of discussion and consolidation, finally becoming known as “Ecological Urbanism” in 2007. Susan Hagan, in an essay for *The Architectural Review*, explains:

The goal of Ecological Urbanism is to create ‘artificial ecosystem’ cities that achieve the same interdependent efficiencies and life-preserving redundancies as natural ecosystems, turning the current linear pattern of energy-in-one-end/wastes-out-the-other into a loop: wastes become energy. The emphasis on environmental systems is a very different way of thinking about the city: urban sites are seen as locations of, not only demand for but supply of, resources. It is an engineering model, vitally important [21].

Thus, ecological urbanism forces us to reimagine the way in which we understand cities. The environmental degradation seen over the course of human history has taught us that we cannot think of cities as the physical manifestation of the triumph of human culture over nature; in doing so, we create false dichotomies between the built and natural environment that spells destruction for both. For cities to become places that guarantee human survival over extended periods of time, the built environment cannot subsume or obliterate the natural environment; it must be in balance, using resources at a rate that allows us to replenish them and producing waste in a way that allows it to become productive material once more [22]. The philosophy behind ecological urbanism as a development paradigm aligns with that of the Munich School which argues that groups and their basic functions are often the foundation of spatial phenomenon in culturally complex landscapes [23]. Thus, ecological urbanism asks us to see building, maintaining, and living in cities as processes of “adaptation rather than domination, ‘living with’ rather than ‘living over’” [21]. Rueda [22] suggests that new information technologies can help us understand and measure environmental, social, and economic realities and that by planning for three levels of the city–aerial, on the surface of the land, and underground–we can better enact the precepts of ecological urbanism.

Yet, as Reffat [17] reminds us, “Theories about the urban condition are useless without the ability to deploy them.” Therefore, in this article, we argue that for a city to be a viable ecosystem as suggested in the theory of ecological urbanism it must be both habitable and sustainable. Habitability, as a general concept, refers to the quality of a built environment to respond to needs of its human population, while sustainability refers to the viability of a place over time due to the use of resources and the management of waste. We suggest that the precepts of sustainable construction provide apt criteria for the evaluation of habitability and sustainability, providing the means for measuring the application of ecological urbanism in the architecture and urban design of different settlements.

### 4.2 Habitability: Making cities comfortable

Douglas [24] in a 2012 editorial about ecological urbanism argues that “urban ecosystems are a hybrid of natural and man-made elements whose interactions are affected not only by the natural environment, but also by human culture, personal behavior, politics, economics and social organization.” In that sense, the purpose of city is to be a place for human beings to live, realize their daily activities, and feel fulfilled while doing so. Therefore, in this paper, we adopt the concept of habitability to analyze the way in which the built environment responds to and satisfies the needs of human residents, arguing that habitability is the first element needed for a city to be understood as an ecosystem.

The concept of habitability is primarily used in countries like Spain and Colombia for establishing legal regulations and norms regarding construction practices, despite its origins in sociology and social psychology [22,25]. In governmental regulatory application, the concept of habitability is most often used to analyze the interior of a residence or commercial space, the materials used in the construction, and the use of resources within the building.

Habitability is typically characterized as related to “acceptability of the environment” or the “extent that [the environment] accommodates its occupants and is acceptable to them” [26]. Habitability is driven by features that are necessary to sustain life (e.g., environmental protection, functioning life support systems), but it is also affected by the design and functioning of the habitat and elements in the habitat [27]. Yang et al. [28] established a comprehensive index of human settlement environment from the perspective of nature and humanity and analyzed the correlation between human settlement environment and population distribution [29]. Nonetheless, scholars including Mercado et al. [30] and Landázuri et al. [31] have come to believe that an interior-centered definition of habitability is incomplete and have therefore refined the concept by dividing it two: internal habitability, which refers to the architectural conditions of buildings as individual units, and external habitability, which considers the way buildings are integrated into the built and natural environments that surround them.

As external habitability evaluates the interconnectivity between residence and built environment–i.e. neighborhood, commercial areas [32], urban infrastructures and installations [33]—it permits a deeper analysis that incorporates the study of how these spaces are used by city residents. For example, Villagrán García [34] argues that habitability necessarily implicates the relationship between human beings and space. Landázuri et al. [31], Moreno Olmos [32], and Castro [35] build upon that foundation, considering habitability as the capacity of built spaces to satisfy the physical and emotional needs of the individuals and groups that occupy them. Moreover, according to Arcas-Abella et al., shifting the discussion of habitability from its internal to its external dimension enables the concept to “face social and environmental issues" in a way that allows cities to be understood as “systems of structures and actions” that balance “the satisfaction of social needs and the regulation of the use of environmental resources” to “stimulate strategies for transformation" [36]. External habitability gives us the tools to understand the city as a dynamic system that feeds upon itself.

### 4.3 Sustainability: Making cities viable over time

The second element of ecological urbanism is sustainability, or the ability of a place to survive and thrive over time. Escobar [37] suggests that the concept of sustainability is the result of the problematization of the dichotomous relation between nature and society, which occurred when people began to truly take note of the environmental degradation generated by the process of capitalist development. Current understanding of sustainable cities is based on the 1987 definition of sustainable development offered in the Report of the World Commission on Environment and Development: sustainable development is that “which meets the needs of the present without compromising the ability of future generations to meet their own needs” [38–40]. Since the 1980s, scholars have suggested that sustainable development is the “integrative and holistic process of maintaining a dynamic balance between the needs and demands of people for equity, prosperity and quality of life, and what is ecologically possible” [17,41,42]. Nonetheless, the discourse of the Bruntland Commission and its supporters reproduce a (neo)liberal perspective that can result in the simplistic exercise of economization of nature. From a broader perspective, therefore, sustainability implies balancing social, economic, and environmental considerations to provide for current and future generations in a way that assures healthy growth not only for the human population but also the natural environment [39,43].

The best way to achieve the objective of sustainable development, we argue, is to consider the urban environment as an ecosystem, as proposed in the framework of ecological urbanism. We can think of a city having a metabolism, which uses resources and produces waste, but also as a part of an “ecological feedback loop” [21], which will achieve homeostasis, the balance needed for long-term survival and well-being, when the consumption of resources does not exceed the ability to produce them, and the production of waste does not overwhelm the ability to transform that waste into a new resource. “In a healthy ecosystem in nature,” Hagan explains, “each biotic member of the system gives as well as takes. If cities are to be physically reformed, they can no longer parasitically consume more than they produce, and architects need enough understanding of the ecosystem model to be able to help close the metabolic loop” [21] and realize sustainability. Achieving sustainable development in a way that aligns with the precepts of ecological urbanism also implies addressing the environmental risks that result from the conditions of habitability. Environmental risk can be defined as the possibility of harm occurring from a specific set of conditions, including the magnitude, duration, and frequency of exposure to a phenomenon or event [44]. In 1983, the National Research Council published *The Red Book* which sought to clarify the practice of risk assessment in the U.S. federal government by broadly defining risk and evaluating current approaches to risk assessment. This characterization encompasses both exposure assessment and response definition and serves as an intermediary between risk assessment and risk management and should summarize the major uncertainties in each step of risk assessment [14,45]. Environmental risk management or risk assessment is the process of identifying, selecting and implementing measures to reduce risks to human health and ecosystems [16,46].

### 4.4 Sustainable construction: Making ecological urbanism a reality

To qualitatively and quantitatively measure the degree to which a settlement fulfills the precepts of ecological urbanism, understood as the conjunction of habitability and sustainability, we turn to concept of sustainable construction, which applies the values of sustainable development to the construction industry for the sake of bettering the quality of life of current and future human residents in a territory [17,38,39,43,47–50]. Sustainable construction argues that the careful use of resources and waste management can result in more habitable living conditions, as well as psychological and emotional satisfaction [51]. For Escobar [37], sustainable construction and ecological urbanism, similarly to sustainable development, would be part of the conversation that arises when the problems of understanding nature and society as two separate, non-related entities are made apparent. Nonetheless, discourse is not an objective description of reality, rather it is a “reflection of the struggle to define reality in certain forms and not others.” Inherent to this struggle are the power relations that permeate discourse and will therefore shape various forms of social intervention.

Shelbourn et.al [39] explain that “construction has a significant effect on quality of life: its outputs alter the nature, function, and appearance of the towns and countryside in which people live and work. The construction, use, repair, maintenance and demolition of such infrastructure consume resources and energy and generate waste.” Because construction not only shapes the built environment on an immediate physical level, but also impacts lifeways, quality of life, and the maintenance of environmental resources in the long-term, it is an industry that must also put sustainability first to bring “environmental responsibility, social awareness, and economic profitability objectives to the fore in the built environment and facilities for the wider community” [38,39,50,52,53]. Baloi [43] further suggests that sustainable construction can be considered as “the creation and responsible management of a healthy built environment based on resource efficient and ecological principles.” It involves changing the fundamental worldviews underpinning the construction of built environments from “linear to cyclical approaches” [50].

Moreover, this change in perspective implicates understanding sustainability in construction as more than environmental responsibility and resource management, but also as a way of seeing the built environment as an essential part of the economic and social life of a community. Therefore, in 2003, Plessis [41] established a set of four criteria for determining if the built environment of a specific settlement was sustainable. The first criterium is “The physical structure; how the settlement sits within the natural environment and therefore responds to the topography; the spatial relationship between the different parts of the city; and the form of the built environment;” the second refers to “The utilization patterns which are formed by the way the settlement uses its resources and which are described by the infrastructure and services provided;” the third is “The social patterns; how people live, learn and work in, and relate to their settlement, and the opportunities provided by the settlement for meeting these social needs;” and the last is “The operational patterns; how the settlement functions and is managed” [17]. With these criteria, the concept of sustainable construction transcends environmental sustainability to embrace economic and social sustainability, emphasizing the possible value addition to the quality of life of individuals and communities.

Reffat [17] builds upon Plessis ‘s general criteria [41] by establishing specific criteria to meet for each aspect of sustainability: ecological, economic, and social. The criteria are shown in Table 3.

**Table 3:** Objectives for measuring sustainable construction.

SOCIAL	ECONOMIC	ENVIRONMENTAL
Occupant Comfort	Local Economy	Water
Inclusive Environments	Capital Costs	Energy
Access to Facilities	Ongoing Costs	Waste
Participation & Control	Efficiency of Use	Site
Education, Health & Safety	Adaptability & Flexibility	Materials and Components

From [14].

Reffat’s [17] fifteen objectives of sustainable construction dovetail nicely onto the concepts of internal habitability, external habitability, and sustainability. Occupant comfort can be understood as the main indicator of internal habitability, which is supported by the construction materials used in the building of the residence. External habitability encompasses inclusive environments, access to facilities, participation and control, and education, health and safety. Sustainability includes, but is not limited to, access to potable water, clean rivers, waste removal, and the mitigation of environmental risk. In the following analysis, sustainability is analyzed through evaluating the management of contamination and the mitigation of environmental and anthropic risk. Crowding and population density is evaluated as a factor that impacts both internal habitability within a home and the sustainability of a settlement over time.

While the theoretical or ideological principles behind sustainable construction are clear and closely mirror the values put forth in discussions of ecological urbanism, a series of deeper questions that continue to impact the realization of truly sustainable construction remains. Escobar in [54] articulates these questions most clearly; he asks if urban design can be separated from its modernist roots, which are characterized by a lack of concern for long-term consequences and unsustainability, to reorient itself to other philosophical commitments, practices, narrative, and ontological embodiments? Moreover, can changing urban design transform the way human beings deal ontologically with other people and living beings and non-living objects so that we consider the future consequences of our present actions?

## 5. Results

The results here presented are divided in three sections according to the concepts used to define ecological urbanism: internal habitability, external habitability, and sustainability. Each section combines qualitative and quantitative research.

### 5.1 Internal habitability

To assess internal habitability, factors like construction materials, home size, and population density in the residence, access to public services, and satisfaction with one’s residence were evaluated. The primary research shows that the internal habitability of the residences in La Primavera meets minimum standards for shelters as defined by Colombian law, and residents, for the most part, accept the living conditions inside their homes. Nonetheless, the buildings do present grave deficiencies, particularly in terms of access to public services. See Table 4 for details.

**Table 4:** Results: Construction materials.

Construction Materials			
Question	Answer options	Number of responses	Percentage
Indicate the structure of the house (Total of 62 responses used for calculation)	Masonry	34	54.8%
	Stilt House	11	17.7%
	Framed Structure	9	14.5%
	Prefabricated	7	11.3%
	Load Bearing Walls	4	6.5%
	Emptied Walls	2	3.2%
Materials used in finishing the façade of the house (Total of 72 responses used for calculation; however, people often used more than one material for finishing the façade and, therefore, the number of responses doesn’t add to 72)	Wood	27	37.5%
	Plastered brick	21	29.2%
	Exposed brick	16	22.2%
	Recycled materials	6	8.3%
	Exposed cement blocks	2	2.8%
	Plastered cement blocks	1	1.4%
	Emptied concrete	1	1.4%
	Prefabricated	1	1.4%
	Tapia	1	1.4%
Roofing Material (Total of 72 responses used for calculation)	Zinc	44	61.1%
	Asbestos cement	13	18.1%
	Clay Teja tiles	10	13.9%
	Flagstone	5	6.9%
Would you like to renovate your house? (Total of 72 responses used for calculation)	Yes	50	69.4%
	No	8	11.1%
	No response	14	19.4%
Flooring Material (Total of 72 responses used for calculation)	Mortar	46	63.9%
	Tile	17	23.6%
	Packed Dirt	8	11.1%
	Wood	1	1.4%

As seen in Table 4, the predominant material used in the structure is masonry. However, to fulfill the norm of seismic resistant construction NSR10, residences must have load-bearing walls or support frames to ensure stability. The residents of a house are still exposed to physical vulnerability from collapse if the structure does not have load-bearing walls or support frames, even when a house is made of wood or brick. Thus, as seen in Table 5 below, a masonry structure is assigned only a medium grade in regard to quality, whereas a framed structure is given a high grade because framed structures have load bearing walls. Furthermore, Table 5 shows that the majority of houses were built with asbestos cement or zinc. The use of such materials in a place with the climate and geographic conditions of La Primavera increases the temperature inside the house, directly affecting the internal habitability, as well as negatively impacting the long-term health of inhabitants.

**Table 5:** Weight and percentage: Construction materials.

Question	Material	Quality Level	Number of Responses	Percentage
Flooring Material (20%) (Total of 72 responses used for calculation)	Tile	High	17	23.6%
	Concrete Mortar	Medium	47	65.3%
	Packed Dirt	Low	8	11.1%
House Structure (40%) (Total of 62 responses used for calculation)	Framed Structure	High	11	17.7%
	Masonry	Medium	40	64.5%
	Stilt House	Low	11	17.7%
Materials used in façade of the house (20%) (Total of 72 responses used for calculation)	Brick or cement block	High	40	55.6%
	Adobe mud and wood	Medium	26	36.1%
	Recycled Material	Low	6	8.3%
Roofing Material (20%) (Total of 72 responses used for calculation)	Clay Teja tile	High	10	13.9%
	Flagstone	Medium	5	6.9%
	Asbestos cement or zinc	Low	57	79.2%

The second indicator of internal habitability is house size and population density. The research shows that in La Primavera, while house size is not uninhabitable, there are significant concerns in relation to overcrowding and having enough space to adapt a home for multiple functions. Table 6 lists the number of people per house and calculates population density per household. There is a correlation between house location and house size: the houses in high-altitude zones, further away from the river, are typically the largest, and generally belong to the earliest residents.

**Table 6:** Assessment of house size and population density in La Primavera.

Question	Grade	Answer	Average
Area of the house (m^2^)	Average (Arithmetic mean)	60 m^2^	
	Minimum	12 m^2^	
	Maximum	250 m^2^	
Number of inhabitants per house	Number of people	Number of Houses	Percentage
	2	17	23.6%
	4	14	19.4%
	1	10	13.9%
	3	7	9.7%
	5	7	9.7%
	6	5	6.9%
	7	5	6.9%
	9	2	2.8%
	8	1	1.4%
	10	1	1.4%
	11	1	1.4%
	60	1	1.4%
	74	1	1.4%
Number of family units per house	Number of family units	Number of Houses	Percentage
	1	60	83.3%
	2	9	12.5%
	3	3	4.2%
Does someone in your family unit realize a productive activity at home? (Total of 68 answers for the calculations)	Yes	16	23.5%
	No	52	76.5%
Is there enough space inside your house to meet the needs of everyone in your family unit? (Total of 72 answers for the calculations)	Yes	42	58.3%
	No	30	41.7%

On the surface, when simply looking at the number of family units per residence, overcrowding does not appear to be a grave concern, however, as the table shows, population density is actually quite high. For those whose homes are less than 60 square-meters, having multiple families in a single home or a family of 5 or more people presents serious conditions of overcrowding, based on Colombian norms which specify that a family unit comprised of three individuals needs a minimum area of 60 square-meters to have healthy and habitable conditions in terms of population density. To analyze the implication of the minimum area on the conditions of overcrowding and intrafamilial violence, the area of the house per person should be assessed, considering 42m^2^ as the minimum house size and the average of 10m^2^ of space per person as the desirable condition because of the norms established by Colombian law. Although 83% of the house are occupied by a single family, in terms of being functional multi-use spaces, the residences of La Primavera meet standards of adequacy, but not excellence, and 9.7% of the population lives in reduced spaces that reflect a condition of overcrowding. As shown in Table 7, the majority of houses in La Primavera have an area that ranges between 22m^2^ and 24m^2^, areas that are well below the minimum size established by Colombian law for the house based on social interests.

**Table 7:** Assessment of house size in La Primavera.

Variable	Question	Formula for the calculation	Quality Level Assessment	Number of houses	Percentage
House size	Area of the house (m^2^) (Total of 62 responses used for calculation)	Area > 70m^2^	High	22	35.5%
		42m^2^ ≤ area < 70m^2^	Medium	16	25.8%
		Area < 42 m^2^	Low	24	38.7%

One cannot consider only the number of family units per house when calculating overcrowding. For example, as seen in results presented in Table 8 below, in La Primavera, the majority of houses are occupied by only one family, but the area and the low number of square meters of private space per person show conditions of overcrowding because many of the spaces in the houses serve various functions simultaneously. Approximately 23.5% (16) of families dedicate their dwelling not only to residence, but also to some productive activity (store, food service, etc.). About 14.3% (10) use the common spaces in the house for productive or social activities during the day, but as sleeping quarters at night. Using the same space inside the house for multiple economic, social, and domestic purposes is due to not having access to places that fulfil the spatial needs related productive activities or income generation outside the house.

**Table 8:** Assessment of population density and overcrowding in La Primavera.

Variable	Question	Answer Description	Quality Level	Number of Responses	Percentage
Population Density	Overcrowding: seeks to identify conditions of overcrowding in the house. Number of family units that occupy the house (Total of 62 responses used for calculation)	No overcrowding: Number of families = number of houses	High	52	83.9%
		Overcrowding: Number of families / house > 1	Low	10	16.1%
	m^2^ per inhabitant per house Calculation is based on dividing the minimum area of the house by the average number of people that comprise the familial group (Total of 62 responses used for the calculation)	Area > 10m^2^/person	High	40	65%
		Area = 10 m^2^/person	Medium	2	3%
		Area < 10 m^2^/person	Low	20	32%
	There is enough space in the house for the needs of everyone in the house. (Total of 72 responses used for calculation)	Yes	High	42	58%
		No	Low	30	42%

The main deficiencies in terms of internal habitability are related to access to public services and amenities. There is no access to formal services; thus, the population has responded by building their own informal service networks. See Tables 9 and 10. Table 9 details which services reach which house; in contrast, Table 10 shows the number of households that have access to more than one public service.

**Table 9:** Assessment of access to public services and amenities in La Primavera.

Question	Answer choices	Number of houses with service or amenity	Percentage
Public services that reach and serve the house (Total of 72 responses used for calculation)	Energy	70	97.2%
	Water	66	91.7%
	Gas	24	33.3%
	Sewage	23	31.9%
	Garbage Collection	21	29.2%
	Internet	13	18.1%
	Telephone lines	2	2.8%
Amenities present in the house (Total of 68 responses used for calculation)	Toilet	67	98.5%
	Shower	57	83.8%
	Laundry patio or room	49	72.1%
	Sink	36	52.9%

**Table 10:** Assessment of the number of services that reach each household in La Primavera.

Variable	Question	Description	Quality Level	Number of responses	Percentage
Access to public services (Total of 68 used for calculation)	Energy, Water, Garbage collection, Sewage	Respondents that have 3 or 4 public services	High	16	22%
		Respondents that have access to 2 public serves	Medium	53	74%
		Respondents that have access to 1 service or have no service	Low	3	4%

Coverage is mediocre but, more worryingly, service quality is poor. The quality of these services should be questioned. The qualitative analysis underlines the precarious, and oftentimes informal and jerry-rigged, nature of service delivery and the resulting impacts on the environment and resident health. Even with electricity, a majority of households continue to depend on natural light and ventilation. The second-best service in terms of coverage is water, however, for many residences, the water that comes out of the tap is not potable. For all, the water comes from the local reservoir higher up the hillside and not from municipal treatment plants. There is no public infrastructure for delivering gas to residences. Gas service is individually provided through personal propane tanks. The most worrying deficiencies are the absences of a sewage system and garbage collection. Residents have attempted to address the lack of state support for infrastructure development by building their own informal systems. Yet, these systems are problematic; for example, the informal sewage system collects wastewater and dumps it into the river. However, residents have not been able to build a treatment plant for the water, which results in the contamination of the river and the destruction of the surrounding ecosystem. Furthermore, due to the lack of garbage collection, residents have created local dump sites for trash behind the settlement, but leakage from the dumpsites is carried to the settlement by rain and also contaminates the land, poisoning fields used for agriculture and animal husbandry as well as further impacting the river.

As a result of poor infrastructure and public services, the residents of La Primavera are exposed to multiple sources of contamination. Table 11 below details the sources of contamination that impact the residents of La Primavera daily and the way residents perceive and identify these types of contamination.

**Table 11:** Assessment of sources of contamination in La Primavera.

Types of Contamination			
Question	Answer choices	Number of respondents	Percentage
Types of contamination that affect the house (Total of 70 responses used for calculation)	Offensive Odors	44	62.9%
	Contaminated Waterways	35	50.0%
	Waste Waters	28	40.0%
	None	16	22.9%
	Garbage Buildup	16	22.9%
	Chemical dumping	12	17.1%
	Gas emissions	8	11.4%

**Fig 3:**
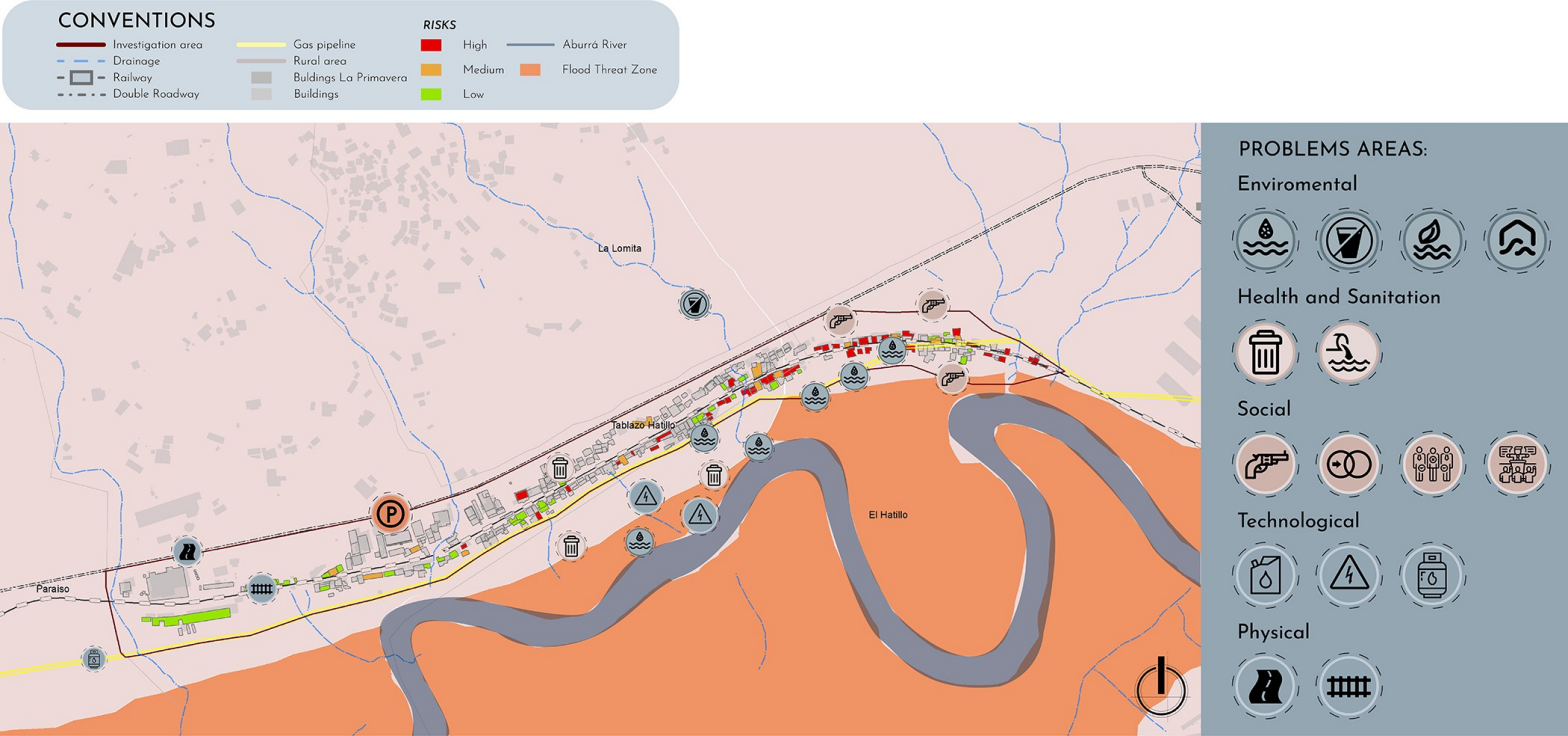
Map of sources of contamination. Source: own preparation.

The contamination caused by the lack of sewage and garbage collection has multiple noticeable effects: respondents identify offensive odors as a type of contamination, as well as contaminated waterways, 4the surface flow of wastewater, and the buildup of garbage as types of contamination affecting their homes. Not only are there multiple sources of contamination, but they are geographically concentrated. Fig 3 offers a map of the sources of contamination found in La Primavera.

The final indicator of internal habitability is the perception of the suitability of the residence for one’s everyday needs. The responses gathered during the primary research were contradictory. Some 58.3% (42) of residents answered that yes, they do consider that their residences meet the conditions needed to carry out their daily lives. In contrast, 41.7% (30) believe that their houses are insufficient to meet their daily needs. For example, respondents often expressed the desire to expand their homes in response to the question “Would you like to renovate your house? Which space? Why?”. Many stated they wanted individual bedrooms, to renovate the kitchen, and to improve the bathrooms.

In conclusion, the findings in relation to internal habitability have shown that the duration of residency in the territory has permitted the population to consolidate their houses and better their conditions of internal habitability in terms of architectural elements. They have established self-managed services to compensate for the lack of state-supported public infrastructure. However, the remarkable initiative of residents still does not compensate for the concerning failure of the state to provide public services and formalize the territory.

### 5.2 External habitability

In La Primavera, the internal habitability of the residences can be considered adequate; however, the external habitability of the surrounding urban system is not. There are grave failures in the provision of public facilities like parks and community centers, physical and institutional infrastructures, and meeting places. Table 12 below shows the facilities that exist near the houses of survey respondents. Respondents could select more than one response; therefore, the percentages do not add. They are used to create a comparison between the percentage of respondents who recognize each public facility.

**Table 12:** Assessment of the type and use of public facilities in La Primavera.

Provision of Public Facilities			
Question	Answer Choices	Number of respondents	Percentage
Public facilities that exist within walking distance of the house (Total of 68 responses used for calculation)	None	36	52.9%
	Educational Facilities	17	25.0%
	Sports Facilities	14	20.6%
	Cultural Center	12	17.6%
	Healthcare Center	9	13.2%
	Social Assistance Center	5	7.4%
Which of these facilities do you use? (Total of 67 responses used for calculation)	None	48	66.7%
	Educational Center	10	13.9%
	Sports zone	6	8.3%
	Community Hall	5	6.9%
	Healthcare Center	3	4.2%
	Hatillo	3	4.2%
	Barbosa	2	2.8%

In contrast with Tables 12 and 13 shows the number of residents who recognize more than one public facility within walking distance of their homes, reflecting the fact that few people have access to vehicular transportation. Combined, the tables show that not only is there a dearth of public facilities, but there is also a lack of knowledge of their existence which results in low rates of usage.

**Table 13:** Assessment of the provision of public facilities in La Primavera.

Variable	Question	Description	Quality Level	Responses	Percentage
Provision of public facilities (Total of 68 respondents used for calculation)	Number of facilities near the settlement	Respondents that identify 4 or 5 public facilities within walking distance of their house	High	5	6.9%
		Respondents that identify 2 or 3 public facilities within walking distance of their house	Medium	8	11.1%
		Respondents that identify 0 or 1 facility within walking distance of their house	Low	59	81.9%

Survey results demonstrate failure of the state to provide essential social facilities in the settlement. The lack of institutional provision of social facilities could be understood as a condition of the informality and illegality of the settlement, conditions used by municipal and departmental authorities to justify their refusal to invest resources in the territory. Nonetheless, the lack of social facilities means that the population must travel to nearby slopes and the *corregimiento* of El Hatillo to satisfy their educational, healthcare, and recreational needs, which in some cases exposes them to increased risk of accidents or long-term health consequences due to not receiving adequate care in time. Residents did identify a local meeting place within the settlement: the community hall. While the hall offers a wooden stage, it presents serious deficiencies in flooring and roofing. In conclusion, in regard to external habitability, different from internal habitability, there were no redeeming factors. Quite simply, there is no access to infrastructure or to public education and healthcare facilities. Furthermore, social or recreational facilities were severely limited and inadequate.

### 5.3 Environmental risk

In La Primavera, the level of non-mitigable environmental risk is problematic. Table 14 details the number of people subject to different types of environmental risk in contrast to their perceptions of that risk. Furthermore, S3 File. Dataset 2. Public Space or Facility Observation Cards shows the technical assessments of the various residences in La Primavera. Each technical observation card includes drawings of the residences as well as the architectural and engineering assessment.

**Table 14:** Assessment of environmental risk in La Primavera and perception of that risk among residents.

Question	Grade	Number of respondents who chose each answer	Percentage
Risk of flooding based on location (Total of 44 responses used for calculation)	High	21	47.7%
	Medium	16	36.4%
	Low	7	15.9%
Perception of the risk of flooding (Total of 72 responses used for calculation)	High	26	36.1%
	Medium	11	15.3%
	Low	35	48.6%
Risk of sinking based on location (Total of 45 responses for calculation)	High	18	40.0%
	Medium	18	40.0%
	Low	9	20.0%
Perception of the risk of sinking (Total of 72 responses used for calculation)	High	19	26.4%
	Medium	11	15.3%
	Low	42	58.3%
Risk of landslides based on location (Total of 44 responses used for calculation)	High	15	34.1%
	Medium	16	36.4%
	Low	13	29.5%
Perception of the risk of landslides (Total of 72 responses used for calculation)	High	18	25.0%
	Medium	9	12.5%
	Low	45	62.5%

**Fig 4:**
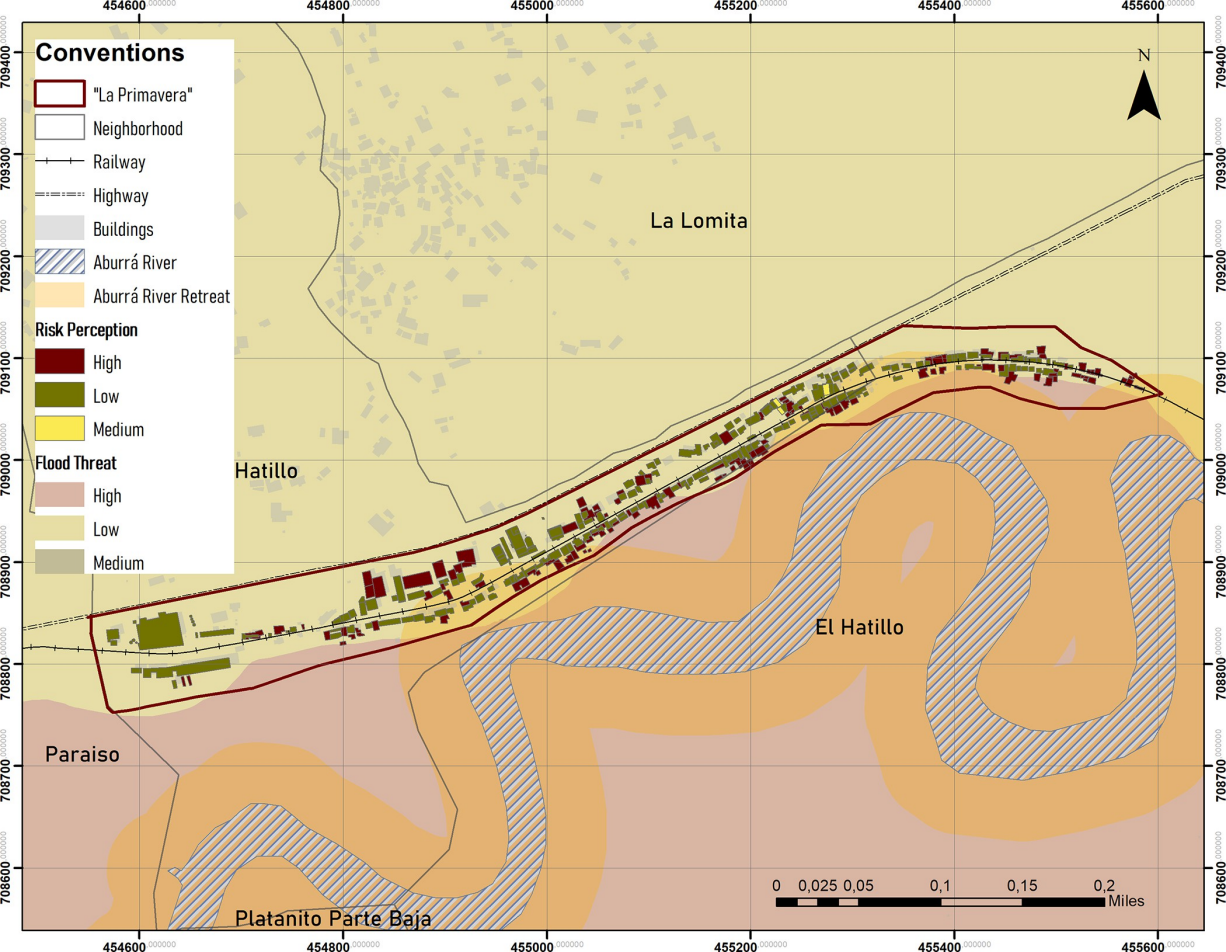
Map of the level of environmental risk in La Primavera. Source: Own preparation.

The perception of risk among the population differed significantly from the risk estimated in the technical visit. While the risk of flooding is well-known throughout the settlement, there are other environmental threats that put the population at greater risk but are less noticed. For example, erosion of the riverbanks from under house foundations is a major problem. The same pattern was seen in relation to landslides. While the perception of the risk is low, the actual level of risk, calculated in the technical assessment is high. Fig 4 offers a map of the levels of risk for houses in the settlement. Houses with low environmental risk levels are marked in green, medium risk yellow, and high risk with red. The map highlights that the houses in the highlands of the settlement have lower environmental risk, as well as having higher conditions of internal habitability. In contrast, risk is concentrated in the lowlands of the settlement, where people have been settled for less time and the conditions of internal habitability are generally lower. The map emphasizes the correlation between risk and habitability.

It is worrisome that 33.3% of the houses in La Primavera are at high risk of flooding, a risk that is perceived as much as it was technically diagnosed in the case study.

If the analysis of external habitability shows great deficiencies in meeting the standards of ecological urbanism, then the findings regarding environmental risk demonstrate the impossibility of speaking of ecological urbanism in informal settlements. The most accessible areas of settlements like La Primavera are often those most at risk from natural threats.

## 6. Discussion

A close reading of the survey results allows us to open the discussion to a more global analysis of the fundamental lines of inquiry: internal habitability, external habitability, and environmental risk. When analyzing these three elements together, we are struck by the permanent tensions between sustainability and habitability in precarious settlements like La Primavera which become obstacles in the way of achieving ecological urbanism.

Regarding internal habitability, at first glance, the prolonged permanence of the population in the settlement of La Primavera appears to have permitted the consolidation of the houses in architectural terms. However, the analysis of the three principal indicators: construction materials, house size, and access to public services highlights the problems that persist in informal settlements. For the indicator of construction materials, although masonry is the predominant material, the absence of load-bearing walls or frames means that the stability of the structure is not guaranteed, which generates a situation of physical vulnerability. With respect to the materials used in flooring, the percentage of packed dirt floors (11%) contributes to increased health risks, which also happens in the case of materials used for the walls. The preponderance of brick or cement blocks is indicative of the level of consolidation of the settlement, as only 8% of the houses analyzed in this study were built using reclaimed or recycled materials. However, the predominance of cement asbestos and zinc in roofing (79.2%) is alarming as these materials generate considerable health risks as well as thermic discomfort.

Moreover, habitability also refers to the quality of a built environment to respond to the needs of its human population, as stated in the theoretical discussion. On the surface, it appears that the long-term processes of settlement consolidation and home improvement in La Primavera have resulted in significant advances in terms of internal habitability. However, in relation to the variable of house size, La Primavera is a clear example of the type of informal settlement where the house size is not uninhabitable, but where there are significant concerns in relation to overcrowding and having enough space to adapt a home for multiple functions. Illustrative of this is how the predominate house size varies between 22m^2^ and 24m^2^, which is smaller than the established legal norm for the minimum area needed for a house to comply with the norms of *Vivienda de Interés Social* (Social Interest Housing), a socially aware housing project that receives government subsidies to help fund construction and reduce the price of the house on the market. Moreover, each space often fulfills multiple functions. Thus, even though the majority of houses are single family homes, they nonetheless can be considered overcrowded because of the low number of square meters per inhabitant. Overcrowding has significant emotional and psychological impacts that can result in long-term health consequences for individuals, as well as generating significant public health concerns.

Furthermore, though 74% of residences in La Primavera have access to two or more public services, the qualitative analysis shows us that quality of services is poor or faulty, and that these services are often supplemented by informal systems which can pose serious health risks or harm the natural environment. Public service provision demonstrates the relationship internal and external habitability, and the case study of La Primavera shows that the lack of high-quality service provision has significant environmental and social impacts, affecting levels of contamination, public health, and overall quality of life.

From a broad understanding, habitability analyzes the relationship between human settlement, environment, and population distribution [29]. An interior-centered definition of habitability is incomplete [30,31], and to truly understand living conditions in a place, both the interior of the house and the exterior built environment of the neighborhood must be analyzed. In the case of La Primavera, external conditions are as problematic and worrisome as internal conditions.

For a place to meet conditions of external habitability, the built environment must foster the relationship between human beings and space [31,32,35]. In La Primavera external habitability, quantified through the provision of public services, is deficient because municipalities often resist the investment of public resources in highly informal zones. The absence of locally provided public facilities forces inhabitants to undertake long and dangerous journeys to access educational facilities, healthcare, recreational spaces, and any other community or public space inhabitants may wish to use. Thus, in La Primavera, there is no social space for community building, social development, or political engagement. Furthermore, the built environment is not conducive to developing or stimulating “strategies for transformation" [36]. As such, the settlement fails to meet the minimum standards to be considered habitable from the perspective of external habitability.

The last line of inquiry used to determine the long-term sustainability and habitability was environmental risk. A marked difference exists between the risk that has been technically characterized and the perception of risk expressed by the community. Roger [46] posits that in relation to environmental risk, identification of threats and the selection and implementation of mitigation strategies are required to reduce the risk to both human health and the landscape. Through this study, we have shown how analysis of internal and external habitability can be used in the identification of environmental risk to understand the impacts of such threats on a community. For example, secondary information shows that the risk of flooding associated with the Aburrá River in the settlement has increased due to climate change. Environmental risk, and the level to which it impacts the settlement and the community, is related to the change in the height and trajectory of the river as the result of climate change and anthropic activities like mining, which occurs directly upstream of the settlement. However, according to the information collected in the external habitability survey, nearly half the individuals surveyed perceive the risk of flooding as low. Moreover, they don’t necessarily acknowledge other sources of environmental risk; the perception of risk is often low even in cases when the technical assessment shows that the risk is high. The preponderance of the perception that environmental risk in La Primavera is low is due, in large part, to the normalization of risk that the inhabitants of informal settlements undergo. Because of the precarious conditions of habitability, inhabitants of informal settlements like La Primavera have developed improvised strategies in reaction to emergency situations.

According to the principles of ecological urbanism, cities must exist in balance, using and replenishing resources at a sustainable rate and transforming waste products into productive material [22]. Only then can cities become places that guarantee human survival and habitable conditions in the long term. The everyday conditions of life in La Primavera and similar settlements exist in complete contradiction to the underlying premises of ecological urbanism. Moreover, these settlements fail to meet the basic requirements for sustainability: they do not meet legal norms for habitability, internal or external, and inhabitants live in contaminated environment under the constant threat of environmental disaster.

## 7. Conclusion

The informal settlement of La Primavera is a typical example of Latin American informal settlements, which, despite their persistence over time (in the case of La Primavera more than 40 years in formation), continue to exist in conditions of physical and environmental precarity that impede the transition to a model of ecological urbanism that has at its base the dual axes of sustainability and habitability, understanding this last as the ability to respond to human needs.

In general terms, in settlements like La Primavera, the conditions of internal habitability expressed in the architectural elements of the residence are the only indicators of the processes of consolidation in the settlement. Furthermore, these architectural elements that improve internal habitability are often the primary or only concern of residents when trying to improve their conditions of habitability. In terms of external habitability, the precarious access to basic infrastructure illustrates a worrying lack of State presence and assistance in the mitigation of risk. In short, in refusing to build infrastructure, the state is ignoring the social and physical needs of these communities, especially those individuals who have been relocated or displaced. In respect to ecological sustainability, informal settlements tend to overwhelm the carrying capacity of the land and increase the likelihood that the population runs the risk of environmental threats like floods, landslides and sinking of the riverbank due to erosion. The interaction of the legal, economic, environmental, and physical conditions of La Primavera make life in the settlement unsustainable in the long run.

The complex and interrelated dynamics of La Primavera are a clear example of the multidimensional character of sustainability. Implementing a model of ecological urbanism implies transcending the architectural realm to understand the relationship between the spatial, biophysical, institutional, and social elements that together enable the intersection of sustainability and habitability. Moreover, scholars and urban planners cannot ignore the local culture, values, and social relations that inform the way people inhabit a specific place and their relationship with the territory.

## Supporting information

S1 File La Primavera habitability interview questionnaire. (DOCX)

S2 File Dataset 1. Survey responses.
(XLSX)

S3 File Dataset 2. Public space or facility observation cards.
(XLSX)
