# Supplementary material for: Ecological urbanism as a condition underpinning urban life for internally displaced peoples in Colombia
Source: PLoS One. 2023 Sep 19;18(9):e0291794. doi: 10.1371/journal.pone.0291794 (PMC10508601; doi:10.1371/journal.pone.0291794)
Supplement: S1 File — (DOCX) [file pone.0291794.s001.docx]

La Primavera Habitability Interview Questionnaire

1. Date of Interview
2. Name of Interviewer
3. Name of Interviewee
4. Age of Interviewee (Years)
5. Time lived in La Primavera (Years)
6. Location
   1. La Primavera, Barbosa
7. Sector
   1. Low altitude / along the riverbank
   2. Middle altitude
   3. High altitude
8. Housing code
9. Latitudinal Coordinates
10. Longitudinal Coordinates
11. Name of the homeowner
12. Legal status of the tenant
    1. Renter
    2. Possessor: Holds title after legalizing 5 or more years of residency
    3. Squatter: Informally hold land due to residency, but not yet legalized
    4. Proprietor: Legal owner from title purchase from the State
    5. Refugee
13. Risk of flooding

|  | High | Medium | Low |
| --- | --- | --- | --- |
| Perception |  |  |  |
| Location |  |  |  |

1. Risk of sinking

|  | High | Medium | Low |
| --- | --- | --- | --- |
| Perception |  |  |  |
| Location |  |  |  |

1. Risk of landslides

|  | High | Medium | Low |
| --- | --- | --- | --- |
| Perception |  |  |  |
| Location |  |  |  |

1. What is the size of your home in square meters (m^2^)?
2. How many family groups live in your house?
3. How many people in your family group work?
4. Does someone in your family group realize a productive activity at home? If so, what space in the house is designated for said activity?
5. Is there enough space inside your house to meet the needs of everyone in your family group?
   1. Yes
   2. No
6. Which public services reach and serve your house?
   1. Electricity
   2. Water
   3. Gas
   4. Phonelines
   5. Garbage collection
   6. Sewage
   7. Internet
7. Do you have any of the following amenities in your home?
   1. Toilet
   2. Sink
   3. Shower
   4. Laundry room or laundry patio
8. Does your house have natural ventilation?
   1. Yes
   2. No
9. Does your house have natural light?
   1. Yes
   2. No
10. What type of flooring do you have in your house?
    1. Dirt floors
    2. Wood
    3. Mortar
    4. Common tile
    5. Ceramic tile
11. Do any of the rooms or spaces in your house have one function during the day and a different function at night?
    1. Yes
    2. No
12. What is the structure of your house?
    1. Stilt house
    2. Load-bearing walls
    3. Masonry
    4. Emptied walls
    5. Framed structure
    6. Prefabricated
13. What materials were used in finishing the façade of your house?
    1. Recycled materials
    2. Wood
    3. Mud or Adobe
    4. Exposed brick
    5. Plastered brick
    6. Exposed cement block
    7. Plastered Cement blocks
    8. Emptied cement
    9. Prefabricated
14. What materials are used in the roofing of your home?
    1. Zinc
    2. Asbestos cement
    3. Clay roof tiles
    4. Flagstone
    5. Ceiling
15. Do you want to renovate your house? Which space? Why?
16. Main facade of the house (Upload picture)
17. Material condition of the house (Upload picture)
18. Zoning of the house (Upload picture)
19. How many floors does your house have?
20. What is the primary type of land usage in your neighborhood?
    1. Residential
    2. Commercial
    3. Services
    4. Industrial
    5. Mixed Use
21. How would you describe the general upkeep and state of your home?
    1. Good
    2. So-so
    3. Poor
22. What is the building typology of your home?
    1. Single family home
    2. Duplex
    3. Three plex
    4. Multifamily
    5. Rural lot with house
    6. Rural lot without a house
    7. Rural suburban
23. Are any of the following facilities present close to your home?
    1. Health Center
    2. Sports Facility
    3. Cultural Center
    4. Social Services
    5. Schools or educational facilities
    6. None of the above
24. Which of the above facilities do you use?
25. Are there any meeting places close to your home?
    1. Yes
    2. No
26. What are the closest meeting places?
27. Which of these meeting places do you use?
28. Do you feel safe going to these meeting places?
    1. Yes
    2. No
29. Are there any emergency assembly points, shelters, or sites for attention should a natural disaster occur?
30. What are these sites of attention?
31. Is there an early warning system?
    1. Yes
    2. No
32. How does the early warning system work? What is its infrastructure?
33. What sources of contamination are you exposed to at home?
    1. Garbage buildup
    2. Offensive smells
    3. Wastewaters
    4. Dirty creeks and streams
    5. Gas emissions
    6. Chemical dumping
    7. None
34. What places do you frequent within La Primavera? What is your route out of La Primavera? What do you do on Saturdays and Sundays? On your typical day, where do you go and what do you do?
35. Who are the most important actors in your community? Do you know of the Nuevo Amanecer Los Meandros Association for Victims and Displaced Peoples? How many Local Action Committee meetings have you attended?
36. What services have you received from the Township of Barbosa? What information have you received from the Township of Barbosa? What information and services have you received on the part of Victim Services agencies?
37. Do you know of some instrument of planning and risk management? What instruments do you know? Do you know the strengths or capacity of response of the members in the face of a risk?
38. Do you know if there have been any interventions along the riverbed? Is your home affected by the riverbed? What benefits or disadvantages has the river brought?
39. Are you displaced? When were you displaced? What did you lose when leaving your place of origin? Were you able to carry out any of your previous activities in your current house? What do you miss? Mention some important characteristics of your place of origin. Did you bring any of those tradition with you? Why did you choose to carry on those traditions here?
40. Are you part of a group or a collective in the community? Which? Do you identify with your community? Do you know the history of La Primavera?
41. Which means of transportation do you generally use to get around?
    1. Metro
    2. Bus / collective
    3. Private vehicle
    4. Walking
    5. Bicycling
    6. Shared vehicle
42. What challenges do you face in relation to your transit and commute around the area?
43. Do you have any final thoughts about La Primavera, the experience of living here, or anything else you would like to share?
    1. Upload audio file
